# Can Intentional Weight Loss Ameliorate Sarcopenia in Individuals with Obesity? A Longitudinal Interventional Study

**DOI:** 10.3390/clinpract12010014

**Published:** 2022-02-17

**Authors:** Hana Tannir, Leila Itani, Dima Kreidieh, Dana El Masri, Marwan El Ghoch

**Affiliations:** 1 Department of Nutrition and Dietetics, Faculty of Health Sciences, Beirut Arab University, P.O. Box 11-5020 Riad El Solh, Beirut 11072809, Lebanon; hana.tannir@bau.edu.lb (H.T.); l.itani@bau.edu.lb (L.I.); d.kraydeyeh@bau.edu.lb (D.K.); dana.masri@bau.edu.lb (D.E.M.); 2 Department of Biomedical, Metabolic and Neural Sciences, University of Modena and Reggio Emilia, 41125 Modena, Italy

**Keywords:** BMI, obesity, sarcopenic obesity, outcome, weight loss

## Abstract

Little remains known regarding the impact of weight loss on sarcopenic obesity (SO), and for this reason we aimed to assess the relationship between the two during a weight management program. Body composition was measured at baseline and six-month follow-up using the Tanita BC-418, and step measurements were obtained daily over a period of six months using an Omron HJ-320 pedometer, in 41 adults of both genders with obesity. The participants were then categorized according to the presence or absence of SO. After a significant weight loss, an improvement in the appendicular skeletal mass (ASM) to weight ratio (24.5 ± 3.5 vs. 26.2 ± 3.6, *p* < 0.01), indicated a decrease in the prevalence of SO by 12.2%. Moreover, these findings were confirmed by logistic regression analysis revealing a significant WL% ≥ 5% combined with an active lifestyle (i.e., ≥8000 steps/day), decreased the risk of SO by 91% (OR = 0.09; 95% CI: 0.02–0.56), after adjusting for age and gender. In conclusion, in a weight management setting, a personalized program for individuals with SO that incorporates new strategies in terms of weight loss and physical activity targets may be adopted to improve the sarcopenia-related index and reduce the prevalence of SO in this population.

## 1. Introduction

Scientific societies primarily concerned with clinical nutrition and obesity recently declared that sarcopenic obesity (SO) [1]—as first defined by Baumgartner [2] and later by others [3,4,5] as represented by the co-existence of excess weight (i.e., an increase in body fat mass deposition) and sarcopenia (i.e., a decrease in muscle mass and strength)—should be considered a clinical research priority [1]. The reasons behind this recommendation stem from the fact that, firstly, patients with SO appear to have a higher risk of cardio-metabolic diseases and psychosocial comorbidities as well as mortality when compared to their counterparts without SO [6,7,8,9,10,11,12,13]. Secondly, recent findings evidenced that SO was associated with decreased energy expenditure [14,15] seemingly related to a negative impact on clinical weight outcomes, namely higher rates of early attrition, and greater difficulties in weight loss maintenance in the long term during weight management programs [16,17].

However, and despite this fact, many uncertainties still surround the clinical management of SO in terms of prevention and/or treatment [18]. Moreover, the impact of intentional weight loss, which occurs in weight management programs on SO remains unclear, as patients do not only lose fat but also muscle mass [19]. In fact, the debate continues on whether weight loss may “ameliorate” or “worsen” sarcopenia-related indices and therefore “decrease” or “increase” the prevalence of SO in this population. In the same direction, little is known concerning the best strategy to incorporate within weight loss programs to manage individuals with SO [20]. As a matter of fact, the available studies on SO prevention/treatment are widely heterogeneous, especially in terms of the studied population (i.e., with or without obesity, clinical or non-clinical sample, young or elderly) in addition to the clarity of interventions (i.e., diet, physical activity, supplementation, pharmacotherapy and their combination, etc.). This was confirmed recently by a systematic review on the effect of nutritional approaches alone or combined with physical activity, where few randomized controlled studies were available [20]. As a consequence of the diverse methodologies employed and the results observed, no clear conclusions were drawn, especially in terms of recommendations [20].

Based on these considerations, the purpose of the current study was to investigate the impact of a weight management program—that incorporates a low-calorie diet based on the Mediterranean diet and an active lifestyle that targets 10,000 daily steps during routine physical activity—on sarcopenia-related indices of SO and its prevalence in a “real-world” clinical setting of patients who are clinically overweight or obese.

## 2. Materials and Methods

Forty-one participants were pooled from a cohort of participants consecutively admitted to the outpatient clinic of the Department of Nutrition and Dietetics at Beirut Arab University (Lebanon) for a weight management program for the treatment of obesity. Participants were deemed eligible if they; were aged ≥18 years, with a body mass index (BMI) ≥25.0 kg/m^2^ and at least one of a number of weight-related comorbidities, were considered indicated for weight loss treatment, had effectively started the treatment, and at least successfully completed the weight loss phase (six months). The program featured a low-calorie Mediterranean diet and active lifestyle. The protocol of the treatment essentially involves a personalized cognitive behavioral treatment (CBT-OB) program designed for patients with obesity described elsewhere [16,21]. The study was approved by the Institutional Review Board of Beirut Arab University (2017H-0035-HS-R-0242), and all participants provided informed written consent.

### 2.1. Demographics and Clinical Status

A questionnaire was administered to patients to retrieve information regarding their medical history and demographic, social and clinical status.

### 2.2. Measures

Body weight and height were measured at baseline and at six-month follow-up using an electronic weighing scale (SECA 2730-ASTRA, Germany) and a stadiometer. The BMI was then calculated according to the standard formula of body weight measured in kg divided by the square of the height in meters. Weight loss percentage (%WL) was calculated from baseline to six-month follow-up.

Body composition was measured at baseline and at six-month follow-up using a segmental body composition analyzer (BC-418, Tanita Corp., Tokyo, Japan) [22,23]. After gender, age, and height information was entered into the device, participants were asked to stand in a stable position in bare feet. The device provided separate body mass readings for different segments of the body, using an algorithm incorporating impedance, age, and height, to estimate the total and regional fat mass (FM) and fat-free mass (FFM) [22,23]. SO was determined at baseline and six-month follow-up, and defined based on the definition of Oh and colleagues, which is a score of less than 23.4 in females and 29.6 in males using the formula (appendicular skeletal muscle mass (ASM))/weight) × 100% [24].

Daily steps were measured over the entire duration of the weight loss phase (six months) by means of a validated commercial grade pedometer (Omron HJ-320; Omron Healthcare Co., Ltd., Kyoto, Japan), considered accurate within ±5% of the measurement criterion, was used to record the total number of steps taken each day, with the device automatically resetting itself at the end of the day and possessing a seven-day memory [22]. The pedometer was placed either in a participant’s trouser pocket or attached to their waistband. Participants were encouraged to wear the pedometer all day for seven days upon awakening and to remove the device only when sleeping or bathing and advised to continue with their usual lifestyle.

### 2.3. Statistical Analysis

Descriptive statistics are presented as means and standard deviations for continuous variables and frequencies and proportions for categorical variables. The Student’s *t*-test was used to compare means and the chi-squared test for independence was employed for categorical variables. Paired *t*-test was used to compare means at baseline and after treatment. Spearman’s correlation analysis was used to assess the correlation between the presence of SO and physical activity and weight loss. Logistic regression analysis was used to assess the effect size of the association measured as an odds ratio. Significance for all tests was set at *p* < 0.05.

## 3. Results

Table 1 presents the characteristics of the study population at baseline. The participants were mostly females (75.6%) with an average age of 43.6 ± 14.6 years. Participants did not differ by education and employment status.

Table 2 shows the anthropometric and body composition variables before and after the weight loss phase (i.e., six months of follow-up), with significant WL% = 9.33 ± 4.87% (range between a max. of 16.76% and min. of 0.37%), translated in a reduction of body weight (92.6 ± 14.8 kg vs. 84.1 ± 15.1 kg, *p* < 0.01), BMI (36.0 ± 5.2 kg/m^2^ vs. 32.8 ± 5.3 kg/m^2^, *p* < 0.01) and BF (39.0 ± 12.5 kg vs. 30.8 ± 11.0 kg, *p* < 0.01).

**Figure 1:**
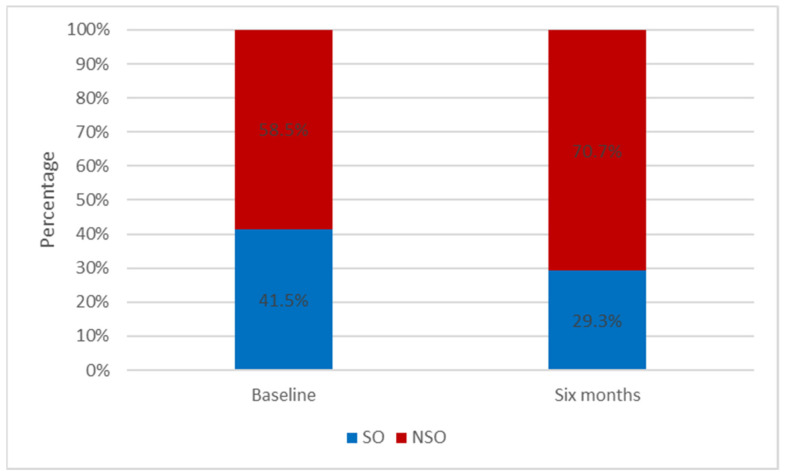
Proportion of patients with SO at baseline and after 6 months of lifestyle modification program.

Moreover, following the six-month lifestyle modification program for weight loss, there was an improvement in the ASM to weight ratio (24.5 ± 3.5 vs. 26.2 ± 3.6, *p* < 0.01) indicating a decrease in the prevalence of SO by 12.2% (41.5% to 29.3%; X^2^ = 23.95, *p* < 0.0001) (Figure 1). Specifically, the least prevalence of SO was observed among those who acquired more steps than the mean daily steps of the population and at the same time lost 5% or more of their body weight loss (walking ≥ 8000 steps/day and WL ≥ 5%) over six months of the lifestyle modification program.

The relationship between the presence of SO and physical activity, as measured by mean daily steps combined with weight loss, was confirmed by correlation analysis (Spearman’s rho = 0.445, *p* = 0.004). Logistic regression analysis revealed that undergoing a lifestyle modification program for six months, including physical activity combined with weight loss (walking ≥ 8000 steps/day and WL% ≥ 5%), decreased the risk of SO after lifestyle modification by 91% (OR = 0.09; 95% CI: 0.02–0.56), after adjusting for age and gender (Table 3).

## 4. Discussion

Our study aimed to provide data on the impact of a short-term weight loss program– based on cognitive behavioral treatment that incorporated a low-calorie diet (i.e., the Mediterranean diet) combined with an active lifestyle—on SO in overweight or obese adults. In turn, one major finding was revealed.

Early weight loss across a period of six months seems to improve the ratio of ASM to weight and reduce the prevalence of SO in a treatment-seeking patient with an overweight or obese condition in an outpatient setting, especially in those who engaged an active daily lifestyle (i.e., >8000 steps/day) and achieved significant weight loss (i.e., WL% > 5%). To date, our study represents one of very few examining the impact of a combined intervention (diet + physical activity) on SO in young adults [25] and not the geriatric population which has been largely discussed in the literature [26,27,28,29]. In fact, our results align with only one earlier study composed of menopausal women with a mean age of 58 years that combined a moderately hypocaloric diet and physical activity of 225 min per week for a duration of 12 months [25]. At the cessation of this study, 35% (vs. 29% in our results) of the sample no longer met the criteria for sarcopenia [25]. However, as concluded by a recent systematic review, despite the fact some interventions are available and can improve SO, overall significance remains limited by a scarcity of data and lack of uniformity in the definition of SO [20]. Further research clarifying optimal treatment options for SO in age classes other than the geriatric population is needed to enable firm conclusions to be drawn, and this was the main aim of our study.

Our study bears certain strengths. Principally, to our knowledge, it is one of few studies assessing the impact of intentional weight loss on SO in patients who are overweight or obese undergoing a weight management program. Furthermore, the longitudinal design, the “real-world” clinical setting of the study, and especially the objective measurement of physical activity in terms of steps over six months on a daily basis should be considered strengths.

However, our study had some limitations. First, our sample included only patients undergoing treatment in an outpatient weight management setting, hence, our findings are not extendable to patients with obesity seeking other treatment modalities (i.e., bariatric surgery, pharmacological treatment, etc.). Secondly, we assessed body composition using an impedance analyzer which, despite being validated, has still not been accepted as a gold standard technique for patients who are overweight and obese [23], although it was also demonstrated as a useful method for detecting SO in this population [30]. Thirdly, no biochemical testing and energy expenditure measurements were conducted; this means we were unable to shed light on the metabolic changes before and after six months of physical activity. Finally, due to the relatively small sample size, these results are preliminary and need further replication. If confirmed, our findings may bear relevant clinical implications, by targeting patients with SO at the baseline, where it may prove useful to implement additional strategies for achieving a certain magnitude of weight loss by adopting a determined active lifestyle with this subgroup of patients which may improve this condition.

## 5. Conclusions

Sarcopenia affects only a subgroup of individuals with obesity, but the reason behind this remains unclear. We speculate that, these individuals are more likely to have an abnormal accumulation of fat (e.g., visceral/ectopic) commonly associated with a significant release of pro-inflammatory cytokines, higher oxidative stress, and reduced anabolic action of insulin-like growth factor-1 (IGF-1) on skeletal muscle, and thus, may lead to sarcopenia [10]. On the other hand, we assume that the opposite can occur, and an intentional WL, reduces BF (i.e., visceral fat), improves inflammation status and mitochondrial function, which ameliorates sarcopenia in this population. In our study, we provide evidence that a WL% of at least 5% and an active lifestyle of 8000 steps per day ameliorate lean mass and the sarcopenia-related index, and reduce the prevalence of SO after short-term weight loss. Undoubtedly, this finding needs to be replicated using larger samples and if confirmed, will provide a new direction for the management of this phenotype (i.e., SO) through personalized strategies for these patients during a weight management program.

## Figures and Tables

**Table 1:** Socio demographic and anthropometric characteristics of the study population (N = 41) *.

Variables	SO at Baseline			
	Non SO N = 24	SO N = 17	Total N = 41	
Age	43.9 (14.0)	43.2 (15.9)	43.6 (14.6)	*p* = 0.881
Sex				X^2^ = 0.397, *p* = 0.529
Male	5 (20.8)	5 (29.4)	10 (24.4)	
Female	19 (79.2)	12 (70.6)	31 (75.6)	
BMI (Kg/m^2^)	33.60 (3.51)	39.44 (5.26)	36.20 (5.16)	*p* < 0.001
Education				X^2^ = 0.897, *p* = 0.344
Lower	15 (62.5)	13 (76.5)	28 (68.3)	
Higher	9 (69.2)	4 (23.5)	13 (31.7)	
Employment				X^2^ = 0.897, *p* = 0.344
Not employed	15 (62.5)	13 (76.5)	28 (68.3)	
Employed	9 (37.5)	4 (23.5)	13 (31.7)	

* The values represent means for continuous variables and (n%) for categorical variables; BMI = body mass index; SO = sarcopenic obesity.

**Table 2:** Anthropometric and body composition variables before and after 6 months of lifestyle modification program (N = 41) *.

Variables	Baseline N = 41	At 6 Months N = 41	*p*-Value
Weight	92.6 (14.8)	84.1 (15.1)	<0.0001
BF	39.0 (12.5)	30.8 (11.0)	<0.0001
BF%	40.1 (6.7)	34.2 (10.2)	<0.0001
BMI (Kg/m^2^)	36.0 (5.2)	32.8 (5.3)	<0.0001
ASM*100/Weight	24.5 (3.5)	26.2 (3.6)	<0.0001
Non SO	24 (58.2)	29 (70.7)	
SO	17 (41.5)	12 (29.3)	

* The values represent means for continuous variables and (n%) for categorical variables; BF = body fat; BMI = body mass index; ASM = appendicular skeletal mass; SO = sarcopenic obesity.

**Table 3:** Odds of having SO after 6 months of lifestyle modification program among individuals who were physically active and had lost at least 5% of baseline body weight (N = 41).

Variables	Multivariate Model	
	Odd ratio	95% CI
Age	0.99	0.93–1.05
Sex		
Male	1.00	
Female	0.43	0.07–2.50
Walking and Weight loss at 6 months		
Walk < 8000 steps and lost below 5%	1.00	
Walk ≥ 8000 steps and lost ≥ 5%	0.09	0.02–0.56

## Data Availability

Are available from the corresponding author on reasonable request.
